# Interaction between psychological variables and response to antalgic treatments in chronic neuropathic pain

**DOI:** 10.1192/j.eurpsy.2026.10340

**Published:** 2026-07-31

**Authors:** C. Perlini

**Affiliations:** 1 Section of Clinical Psychology, Department of Neuroscience, Biomedicine and Movement Sciences, University of Verona; 2 Clinical Psychology Unit, Azienda Ospedaliera Universitaria Integrata (AOUI), Verona, Italy

## Abstract

**Introduction:**

According to a biopsychosocial view of chronic pain, patients’ responses to antalgic treatments are influenced by the interventions themselves, as well as by psychological and contextual factors. This is especially relevant in the use of Spinal cord stimulation (SCS), a minimally invasive surgical technique used to treat people with chronic neuropathic pain (CNP) who have not responded to previous therapies and procedures, by modulating nerve signals.

**Objectives:**

To revise the literature on the relationship between psychological features and response to SCS in CNP, and to discuss some preliminary findings from an Italian sample of patients with CNP recruited at the University Hospital of Verona (Italy).

**Methods:**

Selected patients from the Pain Therapy Center of the University Hospital of Verona undergo a psycho-social assessment at the Clinical Psychology unit before SCS. They were assessed on psychiatric symptoms, personality, presence of trauma, coping strategies, family/friends support, and use of alcohol or substances, with standardized questionnaires. Patients were evaluated at multiple follow-up time points.

**Results:**

Up-to-date literature suggests that several variables may be involved in predicting SCS outcomes, but also points to some gaps in our understanding of failed SCS. To date, 160 patients have been evaluated in our center. Overall, the sample shows elevated presence of somatization, sleep disturbances, depressive and obsessive-compulsive symptoms, together with moderate levels of catastrophizing and a personality profile characterized by health worries and cynicism, despite a high level of perceived self-efficacy and family or social support.

**Conclusions:**

Collected data are presented in the context of the most recent literature, with the aim of strengthening our ability to identify patients at higher risk of SCS failure. The use of a proper pre-SCS assessment also enables the early detection of individuals who may require psychological interventions prior to or following surgery.

**Disclosure of Interest:**

None Declared

